# Antitumor Activity of Small Activating RNAs Induced PAWR Gene Activation in Human Bladder Cancer Cells

**DOI:** 10.7150/ijms.60399

**Published:** 2021-06-16

**Authors:** Kai Yang, Jie Shen, Fu-Qing Tan, Xiang-Yi Zheng, Li-Ping Xie

**Affiliations:** 1 Department of Urology, the First Affiliated Hospital, Zhejiang University School of Medicine, Hangzhou, Zhejiang 310003, P.R. China.; 2 Department of Pharmacy, Traditional Chinese Medical Hospital of Zhejiang Province, Hangzhou, Zhejiang 310006, P.R. China.

**Keywords:** RNA activation, small activating RNA, PAWR, bladder cancer, cell cycle arrest, apoptosis.

## Abstract

Small double-stranded RNAs (dsRNAs) have been proved to effectively up-regulate the expression of particular genes by targeting their promoters. These small dsRNAs were also termed small activating RNAs (saRNAs). We previously reported that several small double-stranded RNAs (dsRNAs) targeting the PRKC apoptosis WT1 regulator (PAWR) promoter can up-regulate PAWR gene expression effectively in human cancer cells. The present study was conducted to evaluate the antitumor potential of PAWR gene induction by these saRNAs in bladder cancer. Promisingly, we found that up-regulation of PAWR by saRNA inhibited the growth of bladder cancer cells by inducing cell apoptosis and cell cycle arrest which was related to inhibition of anti‑apoptotic protein Bcl-2 and inactivation of the NF-κB and Akt pathways. The activation of the caspase cascade and the regulation of cell cycle related proteins also supported the efficacy of the treatment. Moreover, our study also showed that these saRNAs cooperated with cisplatin in the inhibition of bladder cancer cells. Overall, these data suggest that activation of PAWR by saRNA may have a therapeutic benefit for bladder cancer.

## Introduction

Bladder cancer is the fourth most common cancer in men in United States. In 2020, there are estimated 62,100 new cases and 13,050 deaths of bladder cancer in United States 1. Although chemotherapy has revolutionized the treatment of advanced tumors 2, 3, cisplatin-containing combination chemotherapy for metastatic disease only achieve a prolonged median survival of up to 14 months 4 and the associated side effects induced by lack of specificity to tumor cells remain a challenging problem. Therefore, novel therapeutic strategies for the treatment of advanced bladder cancer are urgently required.

Therapeutics based on RNA interference (RNAi) have become powerful and ideal methods for the treatment of many diseases including cancer which are mainly caused by overactive oncogenes because of the high specificity, high efficacy and low toxicity of the RNAi trigger - small dsRNA 5-7. However, there are still many cancers mainly caused by complete inactivation or reduced expression of tumor suppressor genes (TSGs). Recently, new evidence has emerged that synthetic small double-strand RNAs (dsRNAs) could induce sequence-specific transcriptional gene activation of E-cadherin, p21^WAF1/CIP1^ and VEGF by targeting specific regions in their gene promoters 8. This phenomenon was termed RNA-induced gene activation (RNAa) and such dsRNA molecules were termed small activating RNAs (saRNAs) 8. Their observation was supported by another group reporting similar findings 9 and subsequent studies suggests RNAa may be a general and conserved phenomenon of gene regulation 10-17. Moreover, several studies demonstrate that restoration of p21 expression by saRNAs in different cancer cells has been shown to inhibit cell proliferation and tumor growth 18-23. Thus, RNAa holds great promise as an alternative to traditional vector-based systems and would supplement RNA-mediated gene silencing to broaden the gene pool susceptible to therapeutic regulation by small RNAs.

Human PAWR (PRKC apoptosis WT1 regulator) gene, whose other aliases include PAR4 and Par-4, is located in chromosome 12q21 and encodes a leucine zipper domain protein first identified in prostate cancer cells undergoing apoptosis induced by an exogenous insult 24, 25. Functional PAWR protein is essential for apoptosis via diverse cell death pathways 26-28. More importantly, ectopic PAWR over-expression is sufficient to induce apoptosis in most cancer cells *in vitro* and growth inhibition of prostate cancer xenografts in nude mice, but not in normal or immortalized cells 26, 29. Therefore, PAWR is an ideal target and a candidate TSG for RNAa.

Our previous study has reported that dsPAWR-435, a small dsRNA targeting PAWR promoter, can up-regulate PAWR gene expression effectively in human cancer cells 17. In this study, we investigated the antitumor effects of dsPAWR-435 on the bladder cancer cells and found that up-regulation of PAWR by saRNA could not only inhibit cell growth by inducing cell apoptosis and G1-phase arrest, but also cooperate with cisplatin against the growth of bladder cancer cells.

## Materials and Methods

### dsRNA Design and Synthesis

The sequence of dsPAWR-435 (S, 5'- AUA AUA CGG UCU UGU ACU U [dT][dT]-3'; AS, 5'- AAG UAC AAG ACC GUA UUA U [dT][dT]-3') was designed as previously described 17 and the control dsRNA (dsCon: S, 5'-ACU ACU GAG UGA CAG UAG A[dT][dT]-3'; AS, 5'-UCU ACU GUC ACU CAG UAG U[dT][dT]-3') is the same as the dsCon-2 which was specifically designed by Li to lack homology to all known human sequences 8. All dsRNAs were chemically synthesized by GeneChem (Shanghai, China) with dTdT 3' overhangs.

### Cell Culture and Transfection

The human bladder cancer cell line T24 and 5637 were obtained from the Shanghai Institute of Cell Biology, Chinese Academy of Science. The cells were cultured in RPMI 1640 medium supplemented with 10% heat-inactivated fetal bovine serum, penicillin (100 U/mL), and streptomycin (100 mg/L) in a humidified atmosphere containing 5% CO_2_ maintained at 37^o^C. The day before transfection cells were plated in growth medium without antibiotics at a density of 30-40%. Transfections of dsRNAs were carried out by using Lipofectamine 2000 (Invitrogen, Carlsbad, CA, USA) according to the manufacturer's protocol and lasted for 24, 48 or 72 hours. Cell images were taken by using a phase-contrast microscope at 100× magnification (Olympus, Japan).

### Cell growth/viability assay

Proliferation of cells was determined by the (3-(4,5-dimethylthiazol-2-yl)-2,5-diphenyltetrazolium bromide (MTT) assay. Approximately 3000 to 8000 (depending on how long they would be cultured) cells were plated in each well of a 96-well plate. After overnight incubation, the cells were treated with the appropriate dsRNA (mock, 50 nmol/l dsCon, or 1-50 nmol/l dsPAWR-435) for 24, 48, or 72 hours, or with the dsRNA (50 nmol/l dsCon or dsPAWR-435) for 24 hours, followed by incubation for 48 hours with or without 1 μg/ml cisplatin. At the various times after treatment, the medium was removed and MTT (20 μl of a 5 mg/ml solution) was added to each well, and plates were incubated at 37°C for 4 hours. After that, the plates were spun in a centrifuge, and the purple-colored formazan precipitate in each well was dissolved in 150 μl of dimethyl sulfoxide. Absorbance was measured at 490 nm in an absorbance reader (MRX II; DYNEX Technologies, Chantilly, VA, USA). The reduction in viability of each group was expressed as a percentage of the mock or dsCon cells, which were considered to be 100% viable.

### Real-time quantitative RT-PCR

Total RNA was extracted from cells using TRIzol (Invitrogen, Carlsbad, CA, USA) and reverse transcribed using oligo(dT) primers. For real-time quantitative RT-PCR, the resulting cDNA was amplified in a real-time PCR system (ABI Prism 7500, Applied Biosystems, CA, USA) using the DNA-binding dye Sybergreen I (Invitrogen, Carlsbad, CA, USA) for detection of PCR products. Values are expressed as fold-difference compared with Mock. Primer sequences for PAWR are 5'-GCCGCAGAGTGCTTAGATGAG-3' (forward) and 5'-GCAGATAGGAACTGCCTGGATC-3' (reverse) and, for GAPDH are 5'-AAGAAGGTGGTGAAGCAGGC-3' (forward) and 5'-TCCACCACCCTGTTGCTGTA-3' (reverse).

### Western blotting analysis

Briefly, cells were harvested at 48 or 72 hours following dsRNAs treatment as described above, washed and lysed with lysis buffer. Protein concentration in the resulting lysate was determined using the bicinchoninic acid protein assay kit (Pierce Biotechnology, Rockford, IL, USA). Appropriate amounts of protein (30-50 µg) were resolved by electrophoresis in 10-15% Tris-glycine polyacrylamide gels and transferred to nitrocellulose membranes. Membranes were blocked and then incubated overnight with the appropriate primary antibody at dilutions specified by the manufacturer. Primary immunoblotting antibodies were purchased from the Cell Signaling Technology, Beverly, MA, USA. The membranes were next washed three times in 15 ml TBST and incubated with the corresponding horseradish peroxidase (HRP)-conjugated secondary antibody at 1:1000 dilution in TBST for 1 hour. After washing three times for 5 minutes each with 15 ml TBST, bound secondary antibody was detected using an enhanced chemiluminescence (ECL) system (Pierce Biotechnology Inc., Rockford, IL, USA).

To determine NF-κB cellular localization, nuclear and cytoplasmic proteins were isolated from the cells using a cell fractionation kit (KeyGen, Wuhan, China). NF-κB expression in the nuclear and cytoplasmic compartments was determined by immunoblot analysis as described above.

### Detection of apoptotic cells by flow cytometry

A quantitative assessment of apoptosis was made by determining the percentage of cells with highly condensed or fragmented nuclei. Cells were plated in six-well plates and incubated overnight before treatment (Mock, 50 nmol/l dsCon and 50 nmol/l dsPAWR-435). Cells were harvested at 72 hours after dsRNAs treatment as described above, washed twice with pre-chilled PBS, and resuspended in 100 μl 1× binding buffer at a concentration of 1×10^6^ cells/ml. Double staining with fluorescein isothiscyanate (FITC)-conjugated annexin V and propidium iodide (PI) was performed (Annexin V-FITC Apoptosis Detection Kit; BD Biosciences, San Jose, CA, USA) in accordance with the manufacturer's protocol. Cell apoptosis analysis was performed within 1 hour using flow cytometry (Beckman Coulter FC500 Flow Cytometry System with CXP Software; Beckman Coulter, Fullerton, CA, USA).

### Cell cycle analysis by flow cytometry

Cell cycle analysis was performed using a commercial kit (Coulter DNA Prep™ Reagents Kit; Beckman Coulter, Fullerton, CA, USA). Cells were plated in six-well plates and incubated overnight before treatment (Mock, 50 nmol/l dsCon and 50 nmol/l dsPAWR-435). Following treatment, cells were harvested, then washed twice with pre-chilled PBS and resuspended in 100 μl PBS at a concentration of 1×10^6^ cells/ml. Each cell sample was mixed with 100 μl DNA Prep LPR (contained in Coulter DNA Prep™ Reagents Kit), gently mixed by vortex and incubated in the dark at room temperature (25°C) for 20 minutes. Then each was mixed with 1 ml of stain (DNA Prep Stain; contained in Coulter DNA Prep™ Reagents Kit), gently mixed by vortex and again incubated in the dark at room temperature (25°C) for 20 minutes. Finally, cell cycle analysis was performed within 1 hour using flow cytometry (Beckman Coulter FC500 Flow Cytometry System with CXP Software; Beckman Coulter, Fullerton, CA, USA), and the raw data was analyzed by Multicycle for Windows (Beckman Coulter).

### Statistical analysis

All values are expressed as means ± SD. Statistical significance was compared between treatment groups and controls using Student's *t* test. *P* < 0.05 was considered statistically significant.

## Results

### dsPAWR-435 induces PAWR gene activation in bladder cancer cells

**Figure 1:**
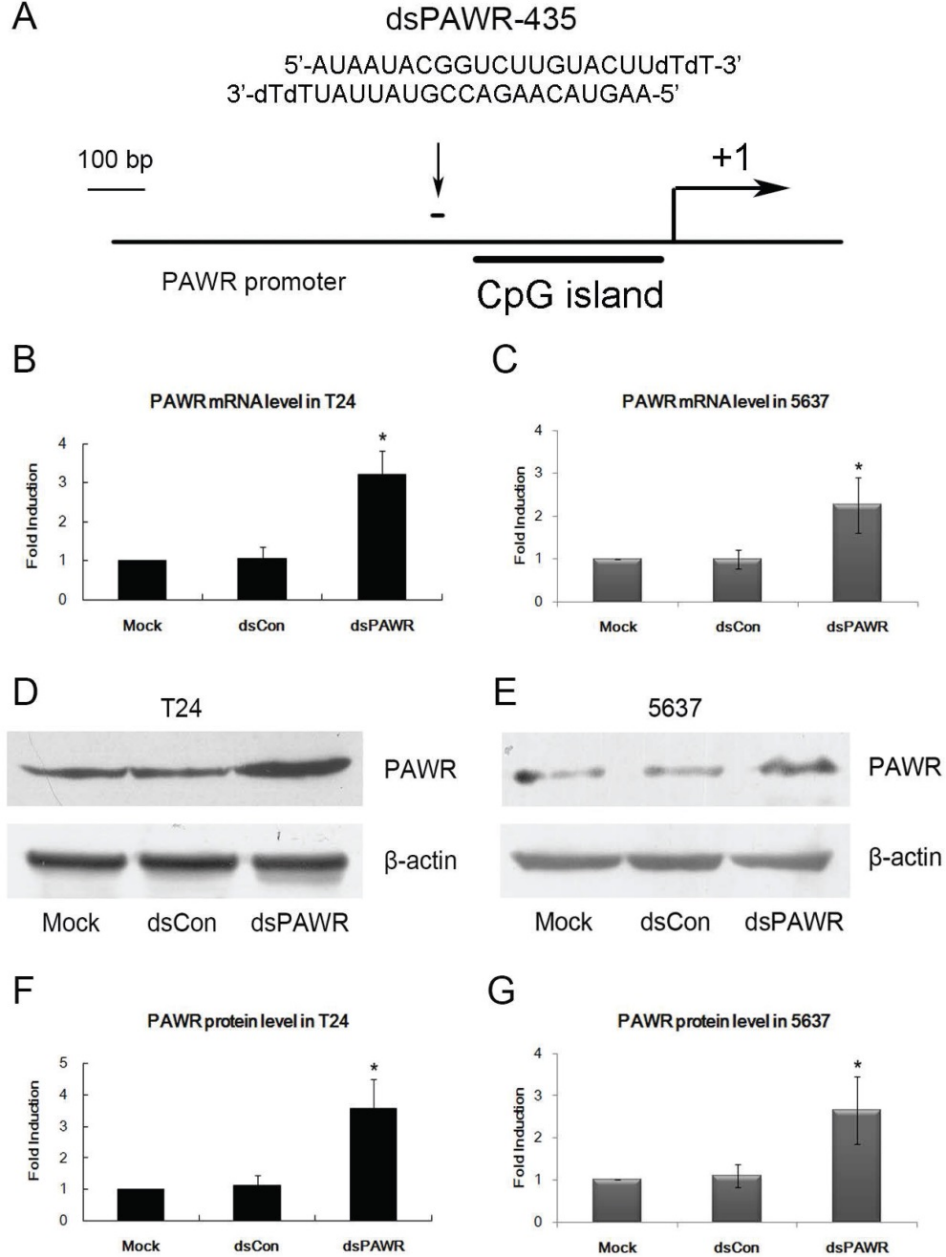
dsPAWR-435 up-regulates PAWR gene expression in bladder cancer cell lines. *: P < 0.05 compared with the mock. (A) A schematic representation of the PAWR promoter with its CpG island, transcription start site, and dsRNA target. T24 (B) and 5637 (C) cells were transfected with 50 nM dsRNAs for 48 hours. mRNA expression of PAWR and GAPDH were detected by real time RT-PCR, and the results were normalized to GAPDH and presented as the mean ± SD of three independent experiments. T24 (D) and 5637 (E) cells were transfected with 50 nM dsRNAs for 72 hours. Induction of PAWR protein expression was detected by Western blot analysis. β-actin levels were also detected and served as a loading control. A representative blot is shown from three independent experiments with identical results. (F, G) Relative protein level was determined by quantifying Western blot membrane band intensity. The PAWR protein expression levels were normalized to β-actin and the results are presented as the mean ± SD of three independent experiments.

A dsRNA targeting the PAWR gene promoter at position -435 relative to the transcription start site (dsPAWR-435) was used to activate PAWR expression as described previously 17 (Fig. 1A). 50 nmol/l (nM) dsPAWR-435 and a nonspecific control dsRNA (dsCon) were transfected into T24 and 5637 human bladder cancer cells and PAWR expression levels were evaluated 48 h and 72 h later. Compared with the Mock and dsCon groups, dsPAWR-435 caused a >2-fold induction in PAWR mRNA levels in both T24 and 5637 cells, respectively (Fig. 1B and C). Induction of PAWR was also confirmed by Western blot analysis (Fig. 1D and E). Elevated levels of PAWR protein strongly correlated to the increase in PAWR mRNA expression in both cell lines (Fig. 1F and G).

### dsPAWR-435 inhibits bladder cancer cell growth and viability

**Figure 2:**
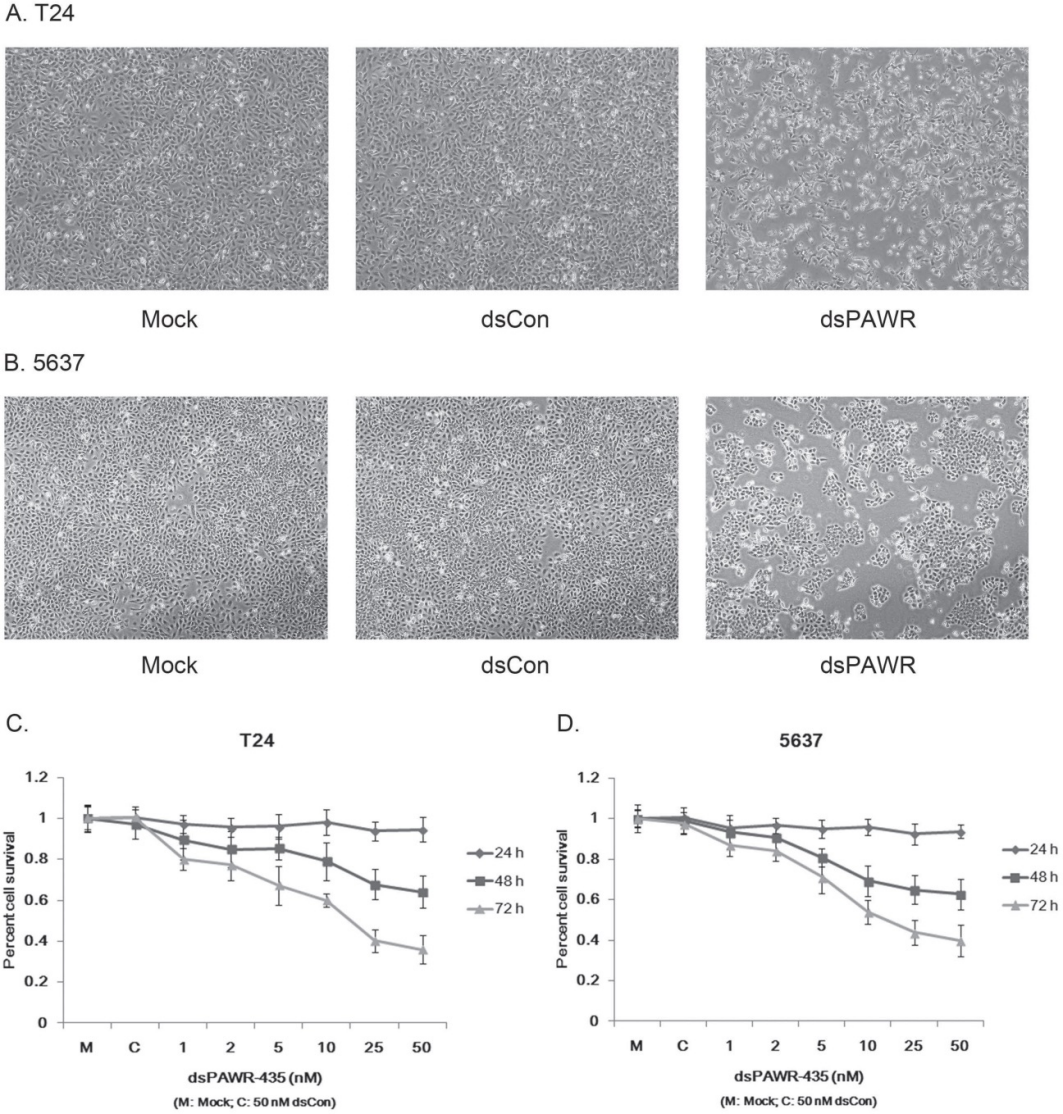
dsPAWR-435 induces growth inhibition of T24 (A) and 5637 (B) bladder cancer cells. Cells were transfected with 50 nM dsRNAs or Mock. Cell images were taken at 24, 48 and 72h after transfection at 100× magnification. dsPAWR-435 transfected cells are less dense and have more dead cells than controls. dsPAWR-435 inhibited the viability of T24 (C) and 5637 (D) cells in a dose-dependent and time-dependent manner, as assessed by the MTT assay. Reduced cell viability was seen after dsPAWR-435 treatment (1 to 50 nmol/l) at 24, 48, and 72 hours. Data are presented as means ± SD (n = 8).

Ectopic PAWR over-expression has been shown to induce growth inhibition in most cancer cells *in vitro* 29. Here we investigated whether the up-regulation of PAWR by saRNA has similar effects on bladder cancer cells. T24 and 5637 bladder cancer cells were transfected by 50 nM dsPAWR-435 and dsCon for 48 or 72 h and dsPAWR-435 transfected cells gradually displayed growth inhibition and cell shrinkage (Fig. 2A & 2B). Moreover, more floated dead cells and evidently decreased cell density were observed in dsPAWR-435 treated group (Fig. 2A & 2B).

**Figure 3:**
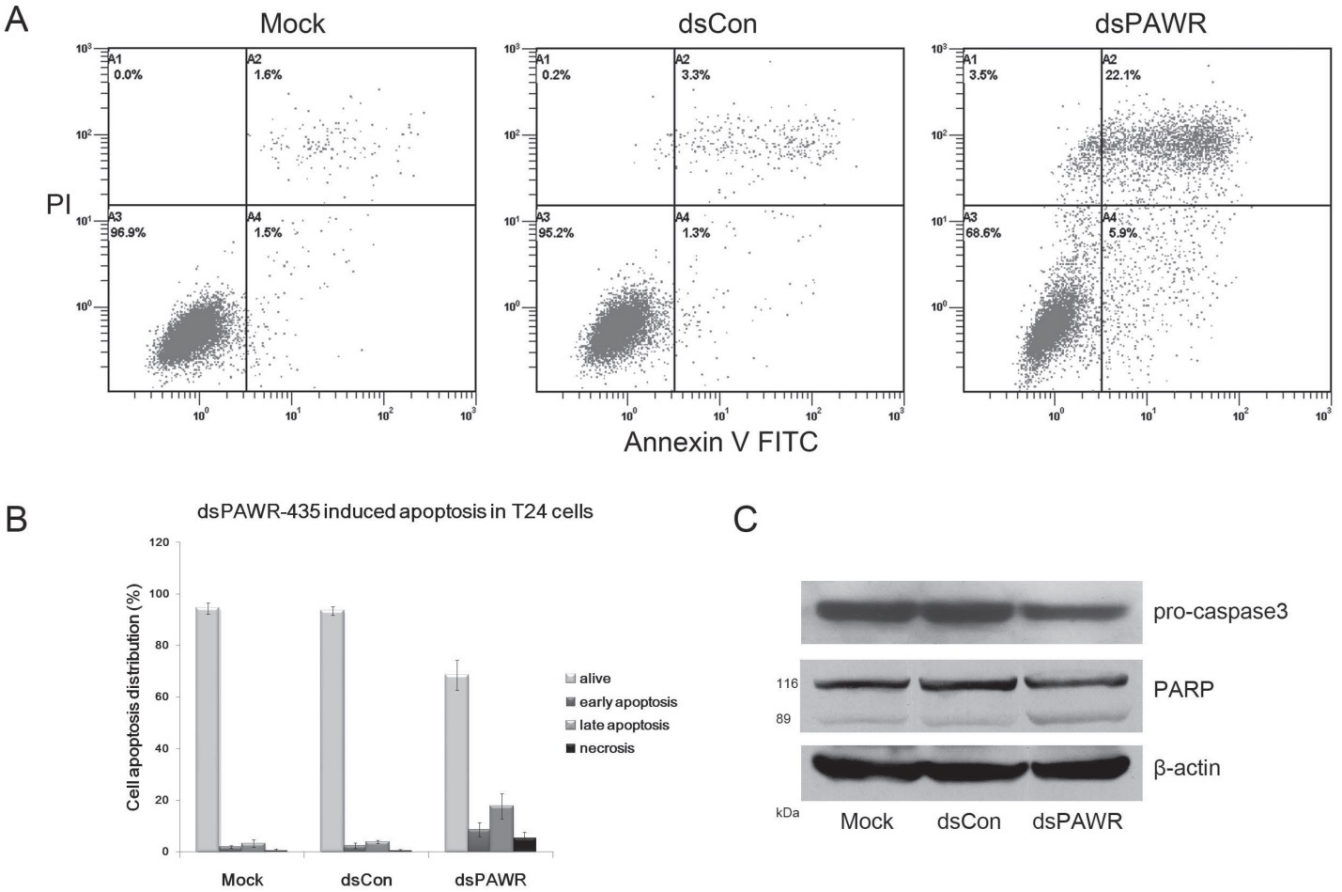
dsPAWR-435 induces cell apoptosis in T24 bladder cancer cells. Cells were transfected with 50 nM dsRNAs for 72 hours. Representative images and blots from three independent experiments with identical results are shown. (A) dsPAWR-435 resulted in apoptosis in T24 cells detected by flow cytometry using a double-staining method with fluorescein thiocyanate-conjugated annexin V and propidium iodide. Annexin V-stained cells indicates the early apoptotic cells, whereas Annexin V + propidium iodide-stained cells are the late apoptotic cells. (B) Flow cytometry data was analyzed to compare cell apoptosis populations (means ± SD from three independent experiments). Percentages of alive, apoptotic and necrotic cells are shown respectively. (C) dsPAWR-435 treatment activated caspase-3 and poly (ADP-ribose) polymerase (PARP) in T24 cells.

The effects of dsPAWR-435 on proliferation and viability of human bladder cancer cells were determined with varying concentrations and times (24 - 72 h) by MTT assay. As shown in Fig. 3, the effects of dsPAWR-435 on cell viability, which were dose and time dependent, occurred within 48 h and at dsRNA concentrations as low as 5 nM. Compared with dsCon transfections, reduction of viability in T24 cells with dsRNA treatment at concentrations of 1 - 50 nM after 48 h ranged from 10.5% to 36.0%, whereas after 72 h ranged from 19.9% to 64.1% (Fig. 2C). Similarly, reduction of viability in 5637 cells treated by dsPAWR-435 after 48 and 72 h ranged from 6.6% to 37.4% and 13.0% to 60.1%, respectively (Fig. 2D).

### dsPAWR-435 induces cell apoptosis in bladder cancer cells

The antitumor ability by ectopic PAWR overexpression is related to its essential role in inducing apoptosis via diverse cell death pathways 26-28. Thus, the relationship between dsPAWR-435-mediated loss of cell viability and cell apoptosis was investigated by flow cytometric analysis labeled with PI and Annexin V and we found that dsPAWR-435 caused evident apoptosis in the T24 cells at 72 h following treatment. As shown in Fig. 3, the ratio of late apoptotic cells (UR quadrant) increased to nearly 20% and the early apoptotic cells (LR quadrant) also increased evidently compared with controls. These data also showed that dsPAWR-435 treatment resulted in not only apoptosis but also tiny cell necrosis, which may be a secondary event in the apoptotic process.

Caspase-3 and poly(ADP-ribose) polymerase (PARP) play central roles in apoptosis. Accordingly, the level of pro-caspase-3 was observed to decrease distinctly in the 50 nM dsPAWR‑435-treated T24 cells at 72 h following treatment (Fig. 3C). Moreover, the 89 kDa cleaved PARP fragment was detected in the dsPAWR-435 treated samples. Thus, the significant changes in apoptosis-related proteins affected by dsPAWR-435 treatment confirmed the ongoing apoptosis above and the anti-tumor effects on the T24 human bladder cancer cells.

### dsPAWR-435 induces cell cycle arrest in bladder cancer cells

**Figure 4:**
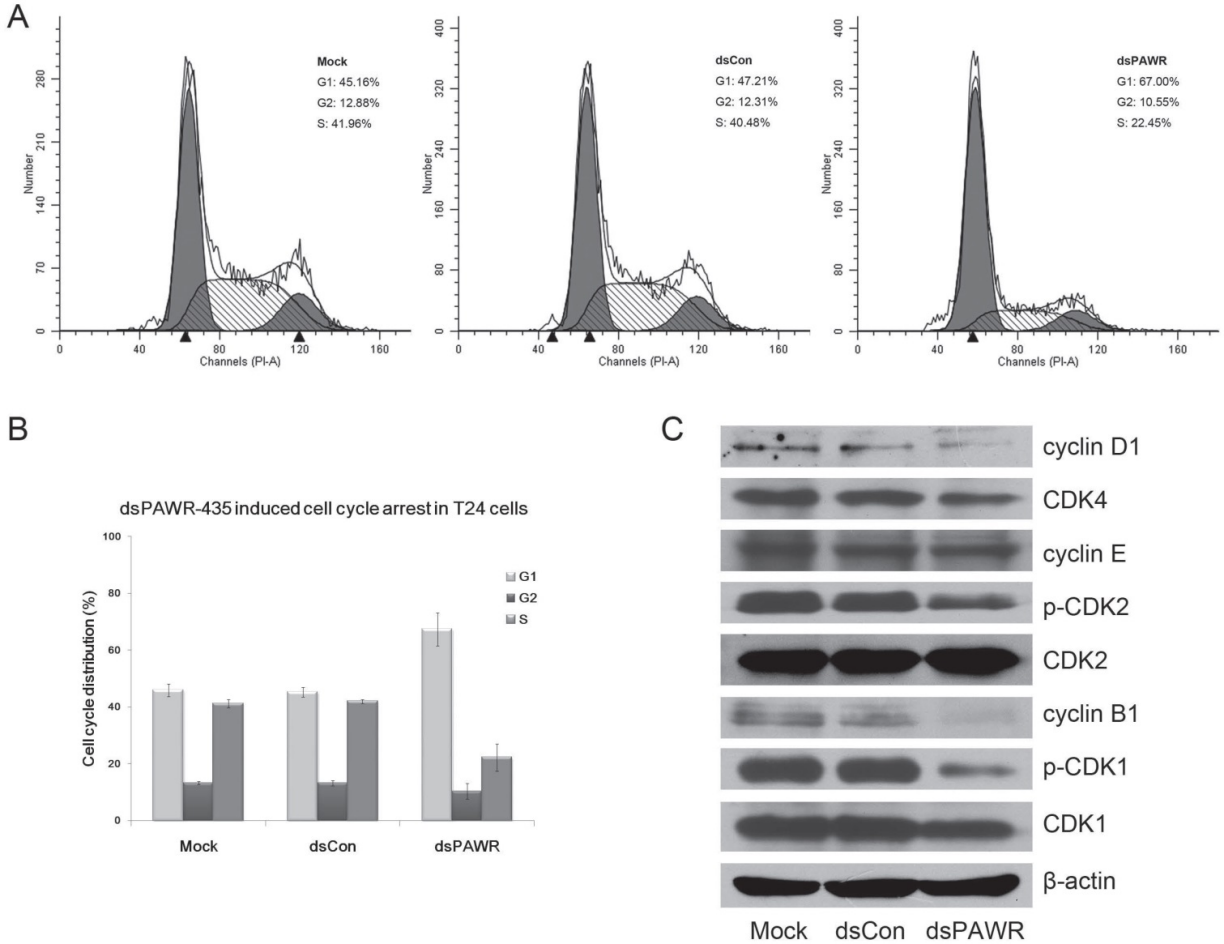
dsPAWR-435 induces G1-phase cell cycle arrest in T24 bladder cancer cells. Cells were transfected with 50 nM dsRNAs for 72 hours. Representative images and blots from three independent experiments with identical results are shown. (A) dsPAWR-435 resulted in G1-phase cell cycle arrest in T24 cells detected by flow cytometry. The sub-G0/G1 cells were not included in the calculations. (B) Flow cytometry data was analyzed to compare cell cycle distribution (means ± SD from three independent experiments). Percentages of G1, G2 and S phase cells are shown respectively. (C) The effects of dsPAWR-435 on cell cycle related proteins in T24 cells.

We next investigated the relationship between dsPAWR-435 mediated inhibition of cell proliferation and cell cycle arrest by flow-cytometric analysis. As shown in Fig. 4, G1-phase arrest in 50 nM dsPAWR-435 treated cells was observed. The G1-phase population of dsPAWR-435 treated cells was about 67% at 72 h following treatment and increased more than 20% at 72 h compared to controls. The increase in cell population in the G1-phase was found to be associated with a concomitant significant decrease in the S-phase population, and less decrease in the G2 phase population was also found.

Then, we examined the expression of several cell cycle-related proteins. Accordingly, interference with the cell cycle in dsPAWR-435 treated T24 cells was associated with decreased expression of cyclin D1 and CDK4. Unexpectedly, we also found decreased expression of cyclin B1 and reduced phosphorylation of CDK2 and CDK1 in dsPAWR-435 treated cells, but no alteration in the expression of cyclin E (Fig. 4C).

### The molecular mechanism related to dsPAWR-435 induced cell cycle arrest and apoptosis

**Figure 5:**
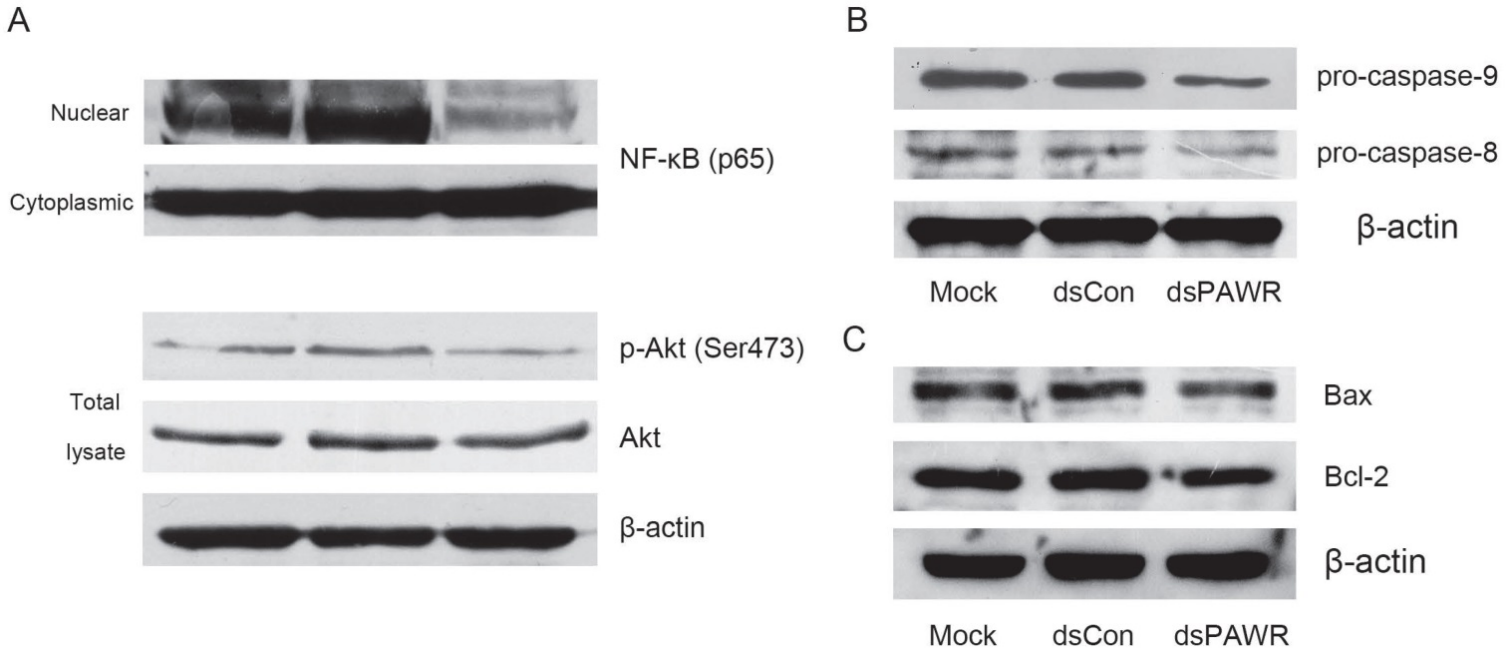
The molecular mechanism related to dsPAWR-435 induced antitumor activity. T24 bladder cancer cells were transfected with 50 nM dsRNAs for 72 hours. Representative blots from three independent experiments with identical results are shown. (A) The nuclear translocation of NF-κB and the phosphorylation of Akt were inhibitied by dsPAWR-435 treatment. (B) The expression of pro-caspase-9 and pro-caspase-8 remarkably decreased in dsPAWR-435 treated T24 cells. (C) Bcl-2 protein was reduced and Bax protein was not affected after dsPAWR-435 treatment in T24 cells.

Previous research has shown that PAWR is an important intersection in the network of tumor suppressors that involves the NF-κB and Akt pathways 30, which are both deregulated during tumorigenesis. Thus, we detected these proteins and found that nuclear translocation of NF-κB and phosphorylation of Akt were inhibited in the dsPAWR-435 treated cells compared with the controls (Fig. 5A), which implied inactivation of these signaling pathways.

To examine which pathway plays a role in the dsPAWR-435 induced cell apoptosis, the cleavage of caspase-8 and -9 was examined. As shown in Fig. 5B, the levels of pro-caspase-8 and -9 were markedly decreased in the 50 nM dsPAWR-435 treated T24 cells at 72 h following treatment, indicating that both extrinsic and intrinsic pathways were active in the dsPAWR-435 treated cells.

PAWR-mediated apoptosis requires downregulation of Bcl-2 levels and PAWR regulates Bcl-2 gene expression through a WT1-binding site in its promoter leading to a decrease in transcription 31, 32. Consistently, the expression of Bcl-2 was found to decrease in the dsPAWR-435 treated cells compared with the controls (Fig. 5C). However, the level of Bax, the pro-apoptotic member of the Bcl-2 family, was not altered after the treatment of dsPAWR-435.

### dsPAWR-435 induced PAWR activation cooperates with cisplatin in inhibition of bladder cancer cells

**Figure 6:**
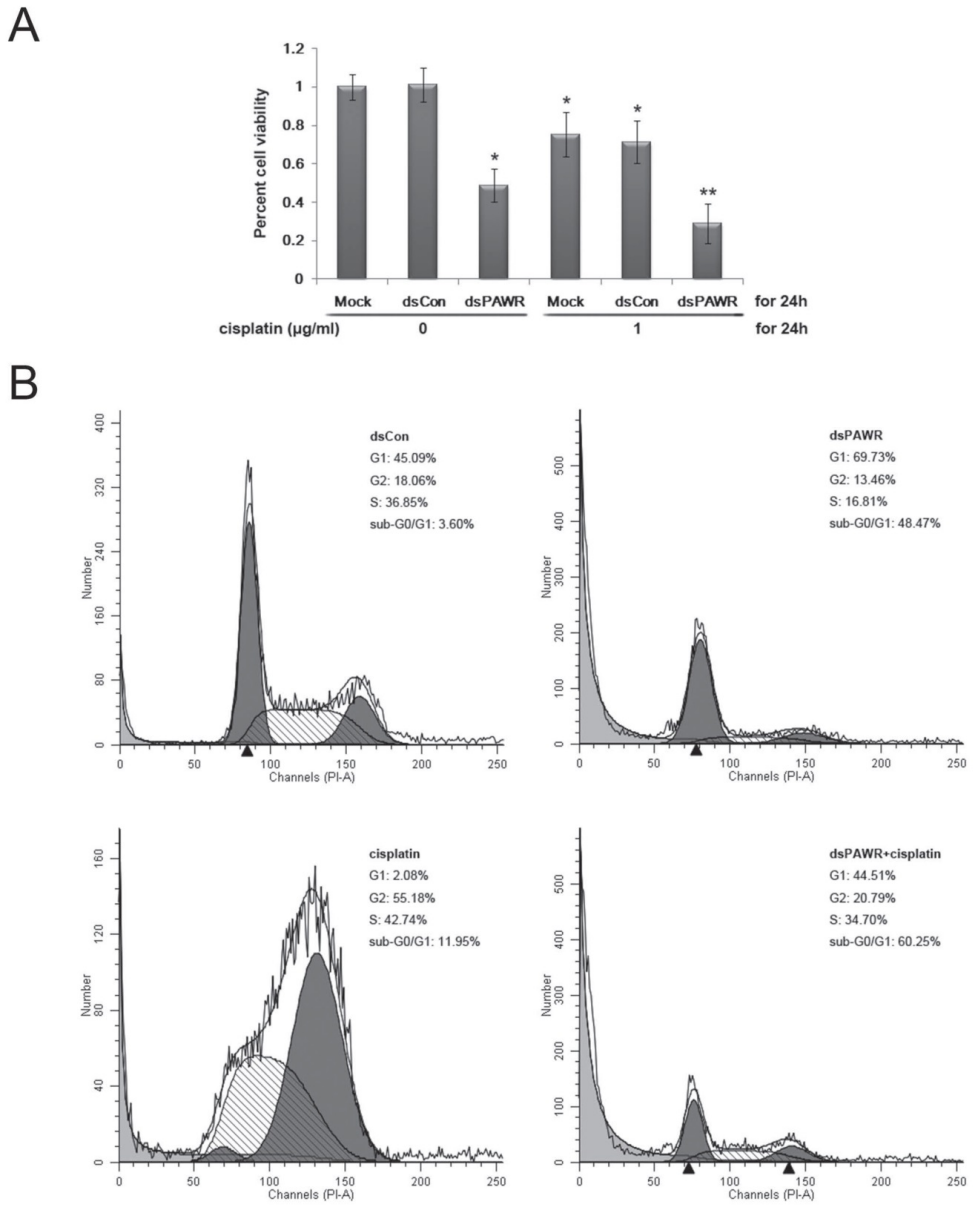
(A) dsPAWR-435 cooperates with cisplatin in inhibiting the viability and growth of T24 bladder cancer cells detected by MTT assay. These data are presented as means ± SD (n = 8). * P < 0.05 versus single treatment with dsCon or Mock. ** P < 0.05 versus single treatment with dsPAWR-435 + cisplatin. (B) dsPAWR-435 cooperates with cisplatin in inducing apoptosis in T24 cells detected by flow cytometry. The percentage of G1, S or G2 phase cells = its number/G1+S+G2 number. The percentage of sub-G0/G1 cells = its number/the number of all cells counted. A representative image is shown from three independent experiments with identical results.

Cisplatin is the main chemotherapy drug for advanced bladder cancer. Thus, we used MTT assay to determine the combined effects of dsPAWR-435 and cisplatin on T24 bladder cancer cells. After transfection with mock, dsCon, or dsPAWR-435 for 24 hours, cells were sub-divided into two groups, and treated or not with 1 μg/ml cisplatin for another 24 hours. The average reduction in cell viability with dsPAWR-435 + cispaltin treatment was 29.0% (Fig. 6A), which was much greater than the reduction obtained with the single treatment of dsPAWR-435 (48.9%) or cisplatin (75.4%).

Both the live cells and the supernatant were then harvested to assess the effects of the treatment above by flow cytometry. Compared with the dsCon-treated group, G1-phase arrest was seen in the dsPAWR-435-treated group and G2-phase arrest was seen in the cisplatin-treated group (Figure 6B). Moreover, there was a significant increase in the sub-G0/G1 population in these two groups (48.47% and 11.95%, respectively), indicating apoptotic cells. When dsPAWR-435-transfected cells were subsequently treated with cisplatin for another 24 hours, the proportion of sub-G0/G1 cells increased to 60.25%, suggesting that the PAWR-targeting dsRNA can co-operate with cisplatin in the inhibition of bladder cancer cells.

## Discussion

RNA activation (RNAa) is an interesting and promising discovery of small RNAs mediated gene up-regulation originally identified in several human cancer cell lines 8, 9. It will offer an alternative to manipulate gene expression potently and specifically if this phenomenon exists in most genes as RNAi and its rules could be deciphered. RNAa thus holds great promise as therapeutics for reactivation of functionally silenced or low expressed TSGs in cancer patients. Our group and others have obtained exciting results that up-regulation of p21 by saRNA could induce cell cycle arrest and apoptosis in human bladder cancer cells 18, 19 and renal cell carcinoma cells 20 *in vitro* and inhibit the growth of xenograft prostate tumor 22 and orthotopic bladder tumor 23 *in vivo*.

Functional PAWR is a TSG essential for apoptosis and a cancer-selective target for cancer therapeutics 33. Depending on the nature of stimulus, apoptosis can occur via two different pathways, extrinsic and intrinsic. Although the two pathways are activated by different mechanisms, they are interlinked by common signaling factors like NF-κB and proteolytic enzymes like caspases 34, 35. Apoptosis by PAWR involves both extrinsic and intrinsic mechanisms. Specifically, PAWR can induce apoptosis by instigating the formation of death inducing signaling complex (DISC) through Fas receptor/Fas Ligand interaction with FADD and causing activation of the Fas/FasL-FADD-Caspase 8 apoptotic death pathway 36. In parallel, the other crucial mechanisms central to apoptosis induced by PAWR is the inhibition of NF-κB transcription function 37. NF-κB can inhibit TNF-α induced apoptosis by blocking protein kinase C (PKC) and can subsequently stall nuclear translocation of the p65 (Rel A) subunit. NF-κB regulates several pro-survival genes such as B cell lymphoma-2 (BCl-2), BCl-XL and Al/Bfl1 as well as anti-apoptotic genes such as an X-linked inhibitor of apoptosis (XIAP) 38. PAWR translocates into the nucleus and inhibits NF-κB-mediated cell survival mechanisms. PAWR-mediated apoptosis requires downregulation of Bcl-2 levels and PAWR regulates Bcl-2 gene expression through a WT1-binding site in its promoter leading to a decrease in transcription 31, 32. Moreover, activation of the Akt pathway is a frequent molecular event in human cancer and PAWR can also inhibit Akt and suppress Ras-induced tumorigenesis 39. Therefore, these interacting factors place PAWR as an important part in the regulation of cell survival, implicating its potential as a candidate for RNAa.

Our attempts with dsRNAs targeting the PAWR promoter have successfully induced transcriptional activation of the PAWR gene in human cancer cells 17. Moreover, dsPAWR-433 actually induced growth inhibition and apoptosis of prostate cancer cells, suggesting that the increased PAWR protein by saRNA is physiologically functional and has great potential for the application in cancer therapy 40. In the present study, we demonstrated that another PAWR promoter targeted dsRNA, dsPAWR-435, could potently induce activation of PAWR gene expression in bladder cancer cells. Promisingly, MTT assay and flow cytometric analysis showed that dsPAWR-435 inhibited cell viability in a dose- and time-dependent manner and it was related to apoptotic cell death. Accordingly, activation of PAWR gene expression by dsPAWR-435 not only activated the caspase-8-dependent extracellular apoptotic pathway but also induced the caspase-9-dependent intracellular apoptosis by inhibition of Akt and NF-κB pathways and downregulation of Bcl-2 protein. Then, the activation of caspase-3 plays a central role in apoptosis by cleaving intracellular proteins vital for cell survival and growth, such as PARP 41, 42, leading to the completion of apoptosis in the dsPAWR-435-treated bladder cancer cells. Moreover, flow cytometric analysis also showed the manifestation of G1 phase arrest in dsPAWR-435-treated cells. Our analysis of cell cycle-related proteins showed that in the dsPAWR-435-treated cells, there were significantly decreased expression of both cyclin D1 and cyclin B1 and reduced phosphorylation of both CDK2 and CDK1. Although cyclin D1/CDK4 and cyclin B1/CDK1 were both inhibited, the dsPAWR-435-treated cells exhibit G1 phase cell cycle arrest.

Over the past two decades, novel therapeutic schemes containing different drug cocktails have been developed, with cisplatin occupying a central position in these regimen (for example the GC (gemcitabine + cisplatin) regimen) 43. It is generally accepted that DNA is the preferential and cytotoxic target for cisplatin 44-46. Cisplatin-mediated damage of genomic DNA causes severe cell cycle perturbation and arrest at certain checkpoints, and in the absence of adequate repair, the affected cells undergo cell apoptosis. In the current study, we found that upregulation of PAWR by saRNA could co-operate with cisplatin and enhance its anticancer effects additively via increased cell apoptosis in T24 bladder cancer cells.

Although dsRNA can successfully induce PAWR expression in bladder cancer cells *in vitro*, we have not been able to obtain good results in animal experiments yet, which might be related to the inefficient delivery and instability of dsRNA *in vivo*. We have tried chemical modification of dsRNA and plan to use more efficient transporter to improve *in vivo* experiments referring to previous literatures 22, 23.

## Conclusions

In conclusion, up-regulation of PAWR by the small double strand RNA dsPAWR-435 in the bladder cancer cells could not only inhibit cell growth by inducing cell apoptosis and G1-phase arrest, but also cooperate with cisplatin against the growth of bladder cancer cells. However, it appears difficult to figure out the definite mechanisms of RNAa due to only a few genes activated and the diversity of the results from different genes. On the other hand, we still have to screen multiple targets in order to activate a particular gene. Regardless, RNAa offers a new approach to enhance endogenous gene expression and holds great promise as a therapeutic for reactivation of functionally silenced or lowly expressed TSGs in cancer patients. Despite the promise, further studies are needed to delineate the exact mechanism of RNAa and develop safe and effective *in vivo* saRNA delivery methods for clinical use.

## Abbreviations

RNAa: RNA activation
saRNA: small activating RNA
RNAi: RNA interference
dsRNA: double-stranded RNA
siRNA: small interfering RNA
PAWR: PRKC apoptosis WT1 regulator
TSG: tumor suppressing gene
MTT: 3-(4,5-dimethylthiazol-2-yl)-2,5-diphenyltetrazolium bromide
nM: nmol/l
